# Urinary Proteomics Identifying Novel Biomarkers for the Diagnosis of Adult-Onset Still’s Disease

**DOI:** 10.3389/fimmu.2020.02112

**Published:** 2020-09-04

**Authors:** Yue Sun, Fan Wang, Zhuochao Zhou, Jialin Teng, Yutong Su, Huihui Chi, Zhihong Wang, Qiongyi Hu, Jinchao Jia, Tingting Liu, Honglei Liu, Xiaobing Cheng, Hui Shi, Yun Tan, Chengde Yang, Junna Ye

**Affiliations:** ^1^Department of Rheumatology and Immunology, Ruijin Hospital, Shanghai Jiao Tong University School of Medicine, Shanghai, China; ^2^State Key Laboratory of Medical Genomics, Shanghai Institute of Hematology, Ruijin Hospital, Shanghai Jiao Tong University School of Medicine, Shanghai, China

**Keywords:** adult-onset Still’s disease, urinary proteomics, α-1-acid glycoprotein 1, orosomucoid, biomarker

## Abstract

Adult-onset Still’s disease (AOSD) is a systemic, multigenic autoinflammatory disease, and the diagnosis of AOSD must rule out neoplasms, infections, and other autoimmune diseases. Development of a rapid and efficient but non-invasive diagnosis method is urgently needed for improving AOSD therapy. In this study, we first performed a urinary proteomic study using isobaric tags for relative and absolute quantification (iTRAQ) labeling combined with liquid chromatography–tandem mass spectrometry analysis in patients with AOSD and healthy control (HC) subjects. The urinary proteins were enriched in pathways of the innate immune system and neutrophil degranulation, and we identified that the α-1-acid glycoprotein 1 (LRG1), orosomucoid 1 (ORM1), and ORM2 proteins were highly expressed in patients with AOSD. The elevated urine levels of LRG1, ORM1, and ORM2 were further validated by enzyme-linked immunosorbent assay in active patients with AOSD, disease controls, and HC subjects. Receiver operating characteristic curves showed that the areas under the curve of LRG1, ORM1, and ORM2 were 0.700, 0.837, and 0.736, respectively (all *p* < 0.05). Furthermore, we found that the urine levels of LRG1, ORM1, and ORM2 were positively correlated with the systemic score and erythrocyte sedimentation rate and that the urine levels of LRG1 were positively correlated with interleukin 1β (IL-1β), IL-6, and IL-18 levels, whereas the urine levels of ORM1 were positively correlated with the IL-1β level. Together, our study identified novel urinary markers for non-invasive and simple screening of AOSD.

## Introduction

Adult-onset Still’s disease (AOSD) is a systemic, multigenic autoinflammatory disease characterized by cardinal manifestations of fever, arthritis and/or arthralgia, skin rash, sore throat, leukocytosis, and excessive neutrophil proportion, in combination with other symptoms, such as myalgia, pericarditis, pleuritis, and elevated erythrocyte sedimentation rate (ESR), C-reactive protein (CRP), and ferritin levels (1–3). The disease may cause life-threatening complications such as fulminant hepatic failure, pulmonary arterial hypertension, disseminated intravascular coagulation, acute respiratory distress syndrome, and macrophage activation syndrome (1). The pathogenesis of AOSD is complicated and still undetermined. AOSD could be affected by genetic background; for example, human leukocyte antigen (HLA) region-related mutations are closely related to the disease (2, 4). Moreover, a recent report declared that cytomegalovirus infections may be implicated as trigger factors for AOSD (5). Most importantly, macrophage and neutrophil activation–associated inflammatory cytokine storms play a crucial role in the disease progression of AOSD (1–3). Pathogen-associated molecular patterns or danger-associated molecular patterns trigger inflammasome activation and the production of interleukin 1β (IL-1β) and IL-18, further improving the expression of the proinflammatory cytokines tumor necrosis factor α (TNF-α) and IL-6 and the anti-inflammatory cytokines IL-10 and IL-37 (1, 6, 7). Additionally, a markedly high frequency of elevated serum molecules such as alarmins (S100A8/A9 and S100A12), chemokines (C-X-C motif chemokine ligand 9, 10, and 11), and microRNAs, which correlate with disease activity, was noticed in patients with AOSD (8–12).

Although AOSD shares many common manifestations and has been considered the archetype of systemic, non-familial autoinflammatory disorders, the current diagnosis of AOSD must rule out neoplasms, infections, and other autoimmune diseases with similar symptoms (1). Thus, developing a rapid, efficient, and non-invasive diagnostic method is urgently needed for the early diagnosis of AOSD. Urine is an important source for the diagnosis of many diseases, because of its non-invasive nature and simple collection. Some contents of urine samples, including metabolites, circulating DNA, microRNAs, and protein, can serve as biomarkers for renal injury–associated diseases or other disorders such as various carcinomas (13–15). However, the urine protein profiles in patients with AOSD are still unknown and might provide potential urinary biomarkers beneficial to disease diagnosis.

In this study, we performed urinary proteomics to explore the landscape of urinary proteins in patients with AOSD and identified three glycoproteins, α-1-acid glycoprotein 1 (LRG1), orosomucoid 1 (ORM1, alternatively named leucine-rich α-2-glycoprotein 1, AGP1), and orosomucoid 2 (ORM2 or AGP2), as potential non-invasive markers assisting the diagnosis of AOSD. Furthermore, we explored the urinary levels of LRG1, ORM1, and ORM2 in patients with AOSD, rheumatoid arthritis (RA), neoplasms, and infections and healthy control (HC) subjects by enzyme-linked immunosorbent assay (ELISA) and determined the correlation between the urinary levels of LRG1, ORM1, and ORM2 and clinical symptoms of AOSD.

## Materials and Methods

### Patients

A total of 70 patients with active AOSD who visited the Department of Rheumatology and Immunology, Ruijin Hospital, Shanghai Jiao Tong University School of Medicine, from January 2018 to April 2019 were consecutively enrolled in this study. The diagnosis of AOSD was made according to the criteria of Yamaguchi et al. (16) after excluding malignancies, infections, and other autoimmune diseases. Fifty age- and sex-matched HC subjects were enrolled. An additional independent set consisting of 24 patients with RA, 14 patients with sepsis, and a heterogeneous group of 27 patients with neoplastic disorders (all malignant, including 19 gastrointestinal neoplasms, 3 genitourinary neoplasms, 3 breast carcinomas, and 2 lung carcinomas) was used to compare the specificity of urinary proteins in AOSD patients. RA was diagnosed according to the 2010 American College of Rheumatology classification criteria (17). Patients with neoplastic disorders were diagnosed with senior oncologists and confirmed by pathology. All sepsis patients fulfilled the Third International Consensus Definitions for Sepsis and Septic Shock (Sepsis-3) (18). The study was performed in accordance with the Declaration of Helsinki and the principles of good clinical practice. Biological samples were obtained under a protocol approved by the Institutional Research Ethics Committee of Ruijin Hospital (identifier 2016–62), Shanghai, China. Informed consent was obtained from the recruited subjects.

Urine samples were collected from patients with active AOSD before treatment with steroid or synthetic disease-modifying antirheumatic drugs. The clinical characteristics and laboratory values (including blood count; ESR, CRP, rheumatoid factor, antinuclear antibody, and ferritin levels; and liver function tests) of each subject were recorded. AOSD disease activity was assessed according to the systemic disease score method (19). Patients with AOSD who had a fever, and/or an inflammatory arthralgia/arthritis, and/or any suggestive cutaneous lesions, and/or a sore throat were considered to be at an active stage (20). All urine and serum samples were immediately stored at −80°C before use.

### Proteomic and Analysis

**FIGURE 1:**
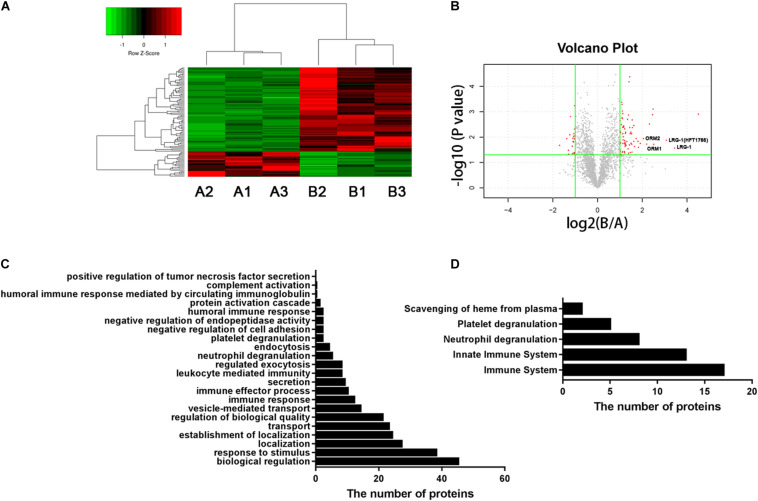
Urinary proteomic analysis in patients with AOSD and HC subjects. **(A)** Heatmap showing the differentially expressed proteins between patients with AOSD (**B** group) and HC subjects (**A** group). **(B)** A volcano plot displaying that LRG1, ORM1, and ORM2 were differentially expressed in patients with AOSD. GO analysis **(C)** and Reactome pathway analysis **(D)** showing the enriched pathways in urine samples of AOSD patients.

To examine the different proteins/peptides from urine between patients with new-onset, treatment-naive AOSD (*n* = 15, from a total of 70 patients) and age- and sex-matched HC subjects (*n* = 15), isobaric tags for relative and absolute quantification (iTRAQ) labeling combined with liquid chromatography–tandem mass spectrometry (LC-MS/MS) analysis were performed by GENECHEM (Shanghai, China) according to a previous report (21). Considering the low abundance of proteins in urine, we pooled urine specimens from five individuals into one sample (combined with different sexes and ages), and each group contained three samples. The UniProt (HomoSapiens_161584_20180123) database was used to blast search the discovered peptides/proteins. The fold change of a protein >2 or <0.5 (*p* < 0.05) considered the difference between the AOSD and HC groups. A heatmap and a volcano plot were generated by R language (the gplots and ggplot packages, respectively). Bioinformatics analysis was performed using the String database^1^. In particular, the biological process of Gene Ontology (GO) analysis and Reactome pathway analysis are shown in Figures 1C,D. Furthermore, 26 secreted proteins listed in Table 1 were enriched in the String database and confirmed by the UniProt database.

**TABLE 1:** Differentially secreted urinary proteins in patients with AOSD.

Accession	Protein name	Description	Fold change	*p*
*Up-regulated proteins*				
P02750	LRG1	Leucine-rich α-2-glycoprotein	10.8792	0.0267
Q68CK4	HMFT1766	Leucine-rich α-2-glycoprotein	8.4340	0.0133
P02763	ORM1	Alpha-1-acid glycoprotein 1	4.5720	0.0188
P19652	ORM2	Alpha-1-acid glycoprotein 2	4.1546	0.0114
A0A1S5UZH5	TXN	Mitochondrial thioredoxin	3.4951	0.0146
P15814	IGLL1	Immunoglobulin lambda-like polypeptide 1	3.3073	0.0114
Q9UIH2	TNFR2	Tumor necrosis factor receptor 2 (fragment)	3.0644	0.0147
B6V6K6	MYOC	Mutant myocilin	2.9448	0.0042
P25311	AZGP1	Zinc α-2-glycoprotein;α-2-glycoprotein 1, zinc	2.8828	0.0191
Q16661	GUCA2B	Guanylate cyclase activator 2B	2.8710	0.0074
A0A0S2Z4R6	SCGB1A1	Secretoglobin family 1A member 1 isoform 1 (fragment)	2.6052	0.0434
D0PNI2	LOX	Lysyl oxidase	2.3426	0.0162
Q5H8C1	FREM1	FRAS1-related extracellular matrix protein 1	2.3186	0.0137
O60234	GMFG	Glia maturation factor γ	2.3094	0.0376
P05161	ISG15	Ubiquitin-like protein ISG15	2.2450	0.0040
Q9UNU2	C4B	Complement protein C4B frameshift mutant (fragment)	2.2166	0.0097
S4R471	AMBP	α-1-Microglobulin/bikunin precursor (fragment)	2.1546	0.0009
Q9UGM5	FETUB	Fetuin-B	2.0441	0.0066
A0A024R930	PRG4	Proteoglycan 4, isoform CRA_a	2.0065	0.0059
A0A024R7R1	HK3	Hexokinase 3 (white cell), isoform CRA_b	2.0055	0.0045
*Down-regulated proteins*				
X6RBG4	UMOD	Uromodulin	0.3856	0.0115
D3DNU8	KNG1	Kininogen-1	0.4045	0.0330
A0A024R6L1	DLK1	Protein delta homolog 1	0.4333	0.0015
G8JLH6	CD9	CD9 antigen	0.4563	0.0228
P02768	ALB	Serum albumin	0.4641	0.0110
Q01469	FABP5	Fatty acid–binding protein 5	0.4843	0.0449

### Enzyme-Linked Immunosorbent Assay

Enzyme-linked immunosorbent assay kits were purchased from Cusabio (Hubei, China) for LRG1 (CSB-E12962h), ORM1 (CSB-EL017237HU), and ORM2 (CSB-E11821h). The urine levels of LRG1, ORM1, and ORM2 in each sample (not pooled together) were detected according to the manufacturer’s instructions. Briefly, 50 μL of the urine samples was added to the previously capture antibody-coated plate, followed by the addition of 50 μL of horseradish peroxidase–conjugated detection antibody and incubation at 37°C for 1 h. After three washes, 90 μL of TMB substrate was added to each well and incubated for 20 min at 37°C. Then, 50 μL of stop solution was added and read immediately at 450 nm using a microplate reader (BioTek Epoch, Winooski, VT, United States).

The serum levels of IL-1β, IL-6, IL-18, and TNF-α were examined by an electrochemiluminescence assay kit from Meso Scale Discovery (MSD, Rockville, MD, United States) according to previous reports (6, 7).

### Statistical Analysis

GraphPad Prism 8.00 software from GraphPad Software Inc. (San Diego, CA, United States) was used to analyze the results in the current study. The KS normality test was used to analyze whether the data fit the parametric contribution. Parametric data are expressed as the mean ± SD, and non-parametric data are expressed as the median with interquartile range. The differences between each group were compared by the non-parametric Mann–Whitney *U* test. The non-parametric Spearman correlation test was performed to analyze the associations between the urinary levels of LRG1, ORM1, and ORM2 and different variables. Receiver operating characteristic (ROC) curves and areas under the curve (AUCs) were determined to evaluate the sensitivity and specificity of the markers. The two-sided principle was carried out during the analyses, and we considered differences to be significant if *P* < 0.05.

## Results

### Urinary Proteomics Analysis Revealed a Unique Panel of Proteins Distinguishing AOSD Patients From HC Subjects

To systematically identify potential biomarkers of AOSD, we collected urine samples and performed proteomics analysis in patients with active AOSD and HC subjects. A total of 92 differentially expressed proteins were identified, of which 71 proteins were up-regulated, and 21 proteins were down-regulated in urine from patients with AOSD compared to those in HC subjects (fold change > 2 or <0.5; *p* < 0.05; Figures 1A,B; Supplementary Table 1). These differentially expressed proteins included 59 functional proteins and 33 proteins with unknown functions. Among the 59 functional proteins, their biological processes displayed the enrichment in the urine of patients with AOSD (Figure 1C). We further analyzed these proteins by Reactome pathway analysis and found that the most enriched pathways were the innate immune system and neutrophil degranulation, in accordance with the pathological features of AOSD (Figure 1D). Interestingly, platelet degranulation-related proteins were also enriched in the urine of AOSD patients (Figure 1D).

Moreover, the GO analysis revealed that 26 proteins were secreted (Table 1), and the levels of the proteins LRG1, ORM1, and ORM2 were highly increased in the urine samples of patients with active AOSD and showed strong protein–protein interactions with each other, as analyzed with the String database (Supplementary Figure 1).

### The Levels of LRG1, ORM1, and ORM2 Increased in Urine From AOSD Patients Validated by Enzyme-Linked Immunosorbent Assay

**FIGURE 2:**
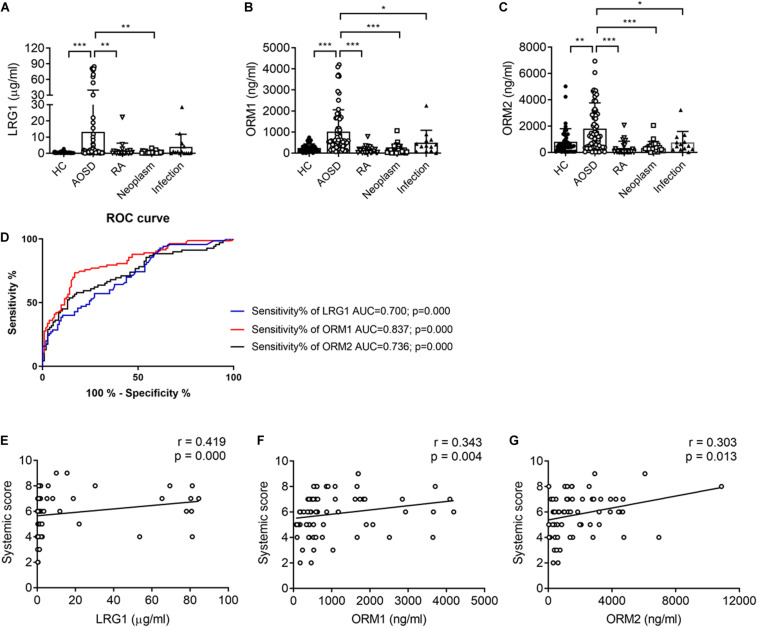
The urinary levels of LRG1, ORM1, and ORM2 were elevated in patients with active AOSD. The levels of LRG1 **(A)**, ORM1 **(B)**, and ORM2 **(C)** in patients with active AOSD (○; *n* = 70), RA (∇; *n* = 24), neoplasm (□; *n* = 27), infection (▲; *n* = 14), and HC subjects (🌑; *n* = 50) were determined by ELISA. **(D)** ROC curves for LRG1 (blue line), ORM1 (red line), and ORM2 (black line) levels to distinguish AOSD from non-AOSD subjects. The correlation between the levels of LRG1 **(E)**, ORM1 **(F)**, and ORM2 **(G)** and the systemic score of AOSD. *p < 0.05; **p < 0.01; and ***p < 0.001.

To confirm the proteomics data, we next validated the protein levels of LRG1, ORM1, and ORM2, the top three increased secreted proteins in the urine samples of patients with AOSD by ELISA. In total, 70 active AOSD patients were enrolled; 50 sex- and age-matched HC subjects were collected as HCs; and 24 patients with RA, 27 patients with neoplasms, and 14 patients with infections were enrolled as disease controls. The clinical and laboratory characteristics of the patients and controls are listed in Table 2. As shown in Figures 2A–C, the urine protein levels of LRG1 were higher in patients with AOSD than in patients with RA or neoplasms and HC subjects, and the urine protein levels of ORM1 and ORM2 in AOSD patients were dramatically higher than those in patients with RA, neoplasms, or infections and HC subjects. Furthermore, we analyzed the diagnostic role of these markers to distinguish AOSD from non-AOSD subjects (including HCs and disease controls). The results in Figure 2D showed that the AUC of LRG1 was 0.700 (*p* = 0.000), the AUC of ORM1 was 0.837 (*p* = 0.000), and the AUC of ORM2 was 0.736 (*p* = 0.000), and a three-panel combined AUC was 0.838 (*p* = 0.000; Supplementary Table 2), suggesting that these three urinary proteins, especially ORM1, could be used as diagnostic markers for AOSD.

**TABLE 2:** Clinical characteristics of patients with AOSD, disease controls, and HC subjects at the time of enrollment.

	Active AOSD (*n* = 70)	RA (*n* = 24)	Neoplasm (*n* = 27)	Infection (*n* = 14)	HC (*n* = 50)
Age (year)	39.6 ± 15.8	51.6 ± 16.8	59.3 ± 10.5	56.0 ± 19.3	37.8 ± 10.3
Gender (female/male)	53/17	15/9	17/10	3/11	37/13
Duration (months)	38.3 ± 65.1	105.2 ± 86.4	NA	NA	NA
*Clinical features*					
Fever	68 (97.1)	0 (0.0)	NA	NA	NA
Sore throat	41 (58.6)	0 (0.0)	NA	NA	NA
Skin rash	59 (84.3)	2 (0.1)	NA	NA	NA
Lymphadenopathy	43 (61.4)	0 (0.0)	NA	NA	NA
Splenomegaly	21 (30.0)	0 (0.0)	NA	NA	NA
Hepatomegaly	3 (4.3)	0 (0.0)	NA	NA	NA
Pericarditis	14 (20.0)	0 (0.0)	NA	NA	NA
Pleuritis	18 (25.7)	0 (0.0)	NA	NA	NA
Pneumonia	28 (40.0)	0 (0.0)	NA	NA	NA
Myalgia	23 (32.9)	0 (0.0)	NA	NA	NA
Arthralgia	60 (85.7)	24 (100.0)	NA	NA	NA
Systemic score	5.8 ± 1.6	1.1 ± 0.3	NA	NA	NA
*Laboratory markers*					
Hemoglobin, g/L	109.3 ± 21.9	117.2 ± 15.7	121.9 ± 17.1	119.1 ± 27.0	NA
Leukocyte, ×10^9^/L	13.0 ± 7.9	6.5 ± 1.9	5.6 ± 2.0	11.2 ± 4.4	NA
Platelet, ×10^9^/L	252.1 ± 99.4	266.5 ± 91.7	201.9 ± 89.1	265.3 ± 95.8	NA
ESR, mm/h	55.8 ± 35.9	45.1 ± 39.5	NA	46.8 ± 36.0	NA
CRP, mg/L	66.5 ± 65.0	25.9 ± 44.9	3.1 ± 1.1	78.5 ± 64.2	NA
ALT, U/L	66.3 ± 112.7	14.7 ± 5.8	29.7 ± 27.1	31.3 ± 16.2	NA
AST, U/L	74.6 ± 172.8	16.6 ± 6.0	30.5 ± 16.8	31.7 ± 16.7	NA
Ferritin, ng/mL	2351.6 ± 3604.6	208.4 ± 281.2	NA	NA	NA
ANA positivity	12 (17.4)	6 (25.0)	NA	NA	NA
RF positivity	1 (1.4)	16 (66.7)	NA	NA	NA

*All values are presented as n (percent) or mean ± SD. AOSD, adult onset Still’s disease; RA, rheumatoid arthritis; HC, healthy controls; ESR, erythrocyte sedimentation rate; CRP, C-reactive protein; AST, aspartate transaminase; ALT, alanine transaminase; ANA, antinuclear antibody; and RF, rheumatoid factor. NA, not available.*

### The Relationship Between the Urinary Protein Levels of LRG1, ORM1, and ORM2 and Disease Activity

**FIGURE 3:**
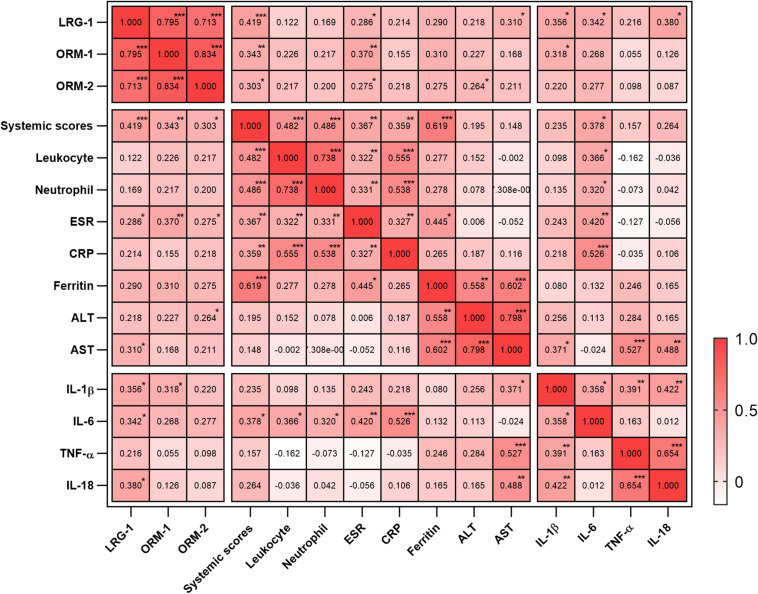
Correlation of urinary LRG1, ORM1, and ORM2 levels with laboratory values in AOSD patients. *p < 0.05; **p < 0.01; and ***p < 0.001.

To evaluate the relationship between the levels of these proteins and disease activity, we analyzed the correlation of the levels of LRG1, ORM1, and ORM2 with the systemic score and laboratory parameters. First, we found that the levels of LRG1 (*r* = 0.419, *p* = 0.000; Figure 2E), ORM1 (*r* = 0.343, *p* = 0.004; Figure 2F), and ORM2 (*r* = 0.303, *p* = 0.012; Figure 2G) were positively correlated with the systemic score. Furthermore, as shown in Figure 3, the levels of LRG1 (*r* = 0.286, *p* = 0.019), ORM1 (*r* = 0.370, *p* = 0.002), and ORM2 (*r* = 0.275, *p* = 0.026) were correlated with ESR. In addition, a positive correlation was found between the levels of LRG1 and aspartate transaminase (*r* = 0.310, *p* = 0.011) and between the levels of ORM2 and alanine transaminase (*r* = 0.264, *p* = 0.032).

### The Association Between the Urinary Levels of LRG1, ORM1, and ORM2 and Clinical Manifestations

Next, we determined the relationship between the typical manifestations of AOSD and the urinary levels of LRG1, ORM1, and ORM2. As shown in Supplementary Table 3, the levels of LRG1 were increased in patients with lymphadenopathy, pneumonia, and pleuritis (*p* < 0.05); the levels of ORM1 were increased in patients with pericarditis and pneumonia (*p* < 0.05).

### The Correlation Between the Urinary Levels of LRG1, ORM1, and ORM2 and Inflammatory Factors

Because cytokine storm is the hallmark of AOSD, we next analyzed the correlation between the urinary levels of LRG1, ORM1, and ORM2 and the levels of serum cytokines including IL-1β, IL-6, IL-18, and TNF-α. As listed in Figure 3, the urinary levels of LRG1 were positively correlated with those of IL-1β (*r* = 0.356, *p* = 0.019), IL-6 (*r* = 0.342, *p* = 0.021), and IL-18 (0.380, *p* = 0.010); the urinary levels of ORM1 were positively correlated with those of IL-1β (*r* = 0.318, *p* = 0.038). Taken together, these results indicated that the urinary levels of LRG1 and ORM1 were associated with the inflammatory conditions of AOSD.

## Discussion

Adult-onset Still’s disease is a systemic autoinflammatory disease, and the pathogenesis of the disease is largely unknown. Thus, using different methods at multiple levels is crucial to understand the landscape of AOSD. In a previous study, we first screened the susceptibility of genetic factors in AOSD using a genome-wide association study and revealed that the SNPs rs3115628 (HLA class I region) and rs9268832 (HLA class II region) were strongly associated with AOSD in the Chinese population (4). Moreover, we identified plasma microRNA profiles using microRNA sequencing in patients with AOSD (12). In the current study, we further explored the urine proteomics of AOSD by iTRAQ-labeling combined with LC-MS/MS analysis. The increased urinary proteins in AOSD were enriched in pathways of the innate immune system and neutrophil degranulation. Hyperactivation of innate immune cells, especially monocytes/macrophages and neutrophils, is the hallmark of AOSD (1, 3). Additionally, the levels of neutrophil granular protein myeloperoxidase and elastase-combined DNA were elevated in patients with AOSD (22). All these results suggested that our proteomic data were coincident with the characteristics of AOSD. We next confirmed the levels of highly enriched secreted proteins LRG1, ORM1, and ORM2 in the urine samples from patients with AOSD by ELISA. Urinary LRG1, ORM1, and ORM2 levels were increased in patients with AOSD compared with those in controls and were positively correlated with disease activity, indicating that the urinary levels of the LRG1, ORM1, and ORM2 proteins might serve as biomarkers for the diagnosis of AOSD. Although previous studies have identified several serum protein markers for AOSD, such as ferritin, inflammatory cytokines (IL-1β and IL-18), anti-inflammatory cytokines (IL-10 and IL-37), and chemokines (CXCL9/10/11) (6, 7, 20, 23), identifying urinary markers will furnish a more non-invasive method for clinical diagnosis.

α-1-acid glycoprotein 1, encoding leucine-rich α-2-glycoprotein 1, is a secreted protein belonging to the leucine-rich repeat (LRR) family. LRG1 is abundant in human serum, mainly produced by hepatocytes, and is involved in the pathogenesis of tumorigenesis and angiogenesis (24, 25). Recently, LRG1 has been recognized as a proinflammatory marker and found to be elevated in patients with ulcerative colitis, type 1 diabetes or RA (26–29). A previous study demonstrated that the serum levels of LRG1 were significantly higher in AOSD patients than in RA patients and HC subjects (30), which was consistent with our results that the urinary levels of LRG1 increased in patients with AOSD compared to those in non-AOSD subjects. Moreover, we demonstrated that the levels of LRG1 were positively correlated with the serum levels of the inflammatory cytokines IL-1β, IL-6, and IL-18 (Figure 3). LRG1 is highly expressed during granulocyte differentiation (31) and facilitates neutrophil differentiation and CD11b expression via G-CSFR signals (32). Further study revealed that LRG1 is a granule protein of neutrophils and cosecreted with lactoferrin (a secondary granule component) when neutrophils are activated (33). Our previous work found that AOSD patients spontaneously released neutrophil extracellular traps including the granule proteins myeloperoxidase and elastase (22), suggesting that neutrophils might be the major source of circulation LRG1 proteins and LRG1 might be involved in the hyperactivation of neutrophils during AOSD pathogenesis.

Orosomucoid 1 and ORM2, encoding ORM1 and ORM2, are plasma proteins related to acute inflammation (34). The ORM proteins are mainly expressed in hepatocytes under stressful conditions, including infections, carcinogenesis, and inflammation (35, 36). The ORM proteins are regulated by TNF-α and IL-1β (36), which were remarkably increased in AOSD and serve as therapeutic targets of the disease (1), suggesting the observed high urinary levels of ORM1 and ORM2 owing to the overproduction of TNF-α or IL-1 in patients with AOSD. Although the function of ORM1 and ORM2 has not yet been well established, the acute-phase protein ORM displays anti-inflammatory features (35). In an ischemic stroke mouse model, ORM inhibited the production of the inflammatory cytokines IL-1β, IL-6, and TNF-α (37). In LPS-induced neuroinflammation, ORM2 attenuated C-C chemokine ligand 4 (CCL4)–mediated activation and migration of microglia (38). ORM proteins could be applied as diagnostic markers for the early events of many pathological states. Elevated urinary ORM levels have been reported as biomarkers for multiple carcinomas and inflammatory diseases such as psoriasis, Crohn disease, and sepsis (39–43). Notably, Park et al. (44) demonstrated that the urinary levels of ORM1 and ORM2 were increased in patients with RA and correlated with disease activity. Interestingly, they revealed that the urinary levels of ORM2 could predict radiographic progression in patients with RA. Arthralgia is a primary symptom of AOSD involving 73.1% of patients with AOSD in China (45) and might progress into arthritis after a long disease duration. Therefore, whether urinary ORM2 could be a predictive biomarker for arthritis in AOSD is a meaningful topic and requires a long-term follow-up study.

It is interesting that the three proteins and the other proteins discovered by proteomic analysis, such as zinc α-2-glycoprotein (AZGP1) and α-1-microglobulin/bikunin precursor (AMBP), are all glycoproteins and associated with inflammatory conditions, indicating an indispensable role of glycoproteins and glycosylation in inflammatory diseases. However, there are some limitations in our study. First, only active AOSD patients were enrolled in the current study, and these patients need follow-up. Second, patients with neoplasms possessed mixed conditions, including different diseases. More specific disease controls should be collected, and the numbers of disease controls need to be enlarged in future studies. Third, the detailed functions and mechanisms of LRG1, ORM1, and ORM2 remain inadequately determined and need future explorations.

Overall, for the first time, we performed a proteomic analysis to analyze the urinary protein profiles of AOSD and unveiled that the urinary levels of LRG1, ORM1, and ORM2 were highly enriched in patients with AOSD and correlated with disease activity and inflammatory indicators. Moreover, the remarkable ROC performance of the ORM1 protein provided a much more convenient, non-invasive approach for the screening of AOSD.

## Data Availability Statement

The datasets generated for this study can be found in the integrated proteome resources (iProX) with project ID of IPX0002355000.

## Ethics Statement

The studies involving human participants were reviewed and approved by the Institutional Research Ethics Committee of Ruijin Hospital (identifier 2016–62), Shanghai, China. The patients/participants provided their written informed consent to participate in this study.

## Author Contributions

YS and JY conceived of the study and participated in its design and coordination. FW and ZZ carried out the ELISA and performed the statistical analysis. JT, YS, HC, ZW, QH, JJ, TL, HL, XC, and HS collected samples and contributed to data acquisition, analysis, and critical review for intellectual content. FW performed the statistical analyses for all the data. YS, JY, YT, and CY drafted the manuscript and revised the manuscript. All authors read, revised, and approved the final manuscript.

## Conflict of Interest

The authors declare that the research was conducted in the absence of any commercial or financial relationships that could be construed as a potential conflict of interest.

## Footnotes

1 https://string-db.org/
