# Supplementary figures and images for: Urinary Proteomics Identifying Novel Biomarkers for the Diagnosis of Adult-Onset Still’s Disease
Source: Front Immunol. 2020 Sep 4;11:2112. doi: 10.3389/fimmu.2020.02112 (PMC7500098; doi:10.3389/fimmu.2020.02112)

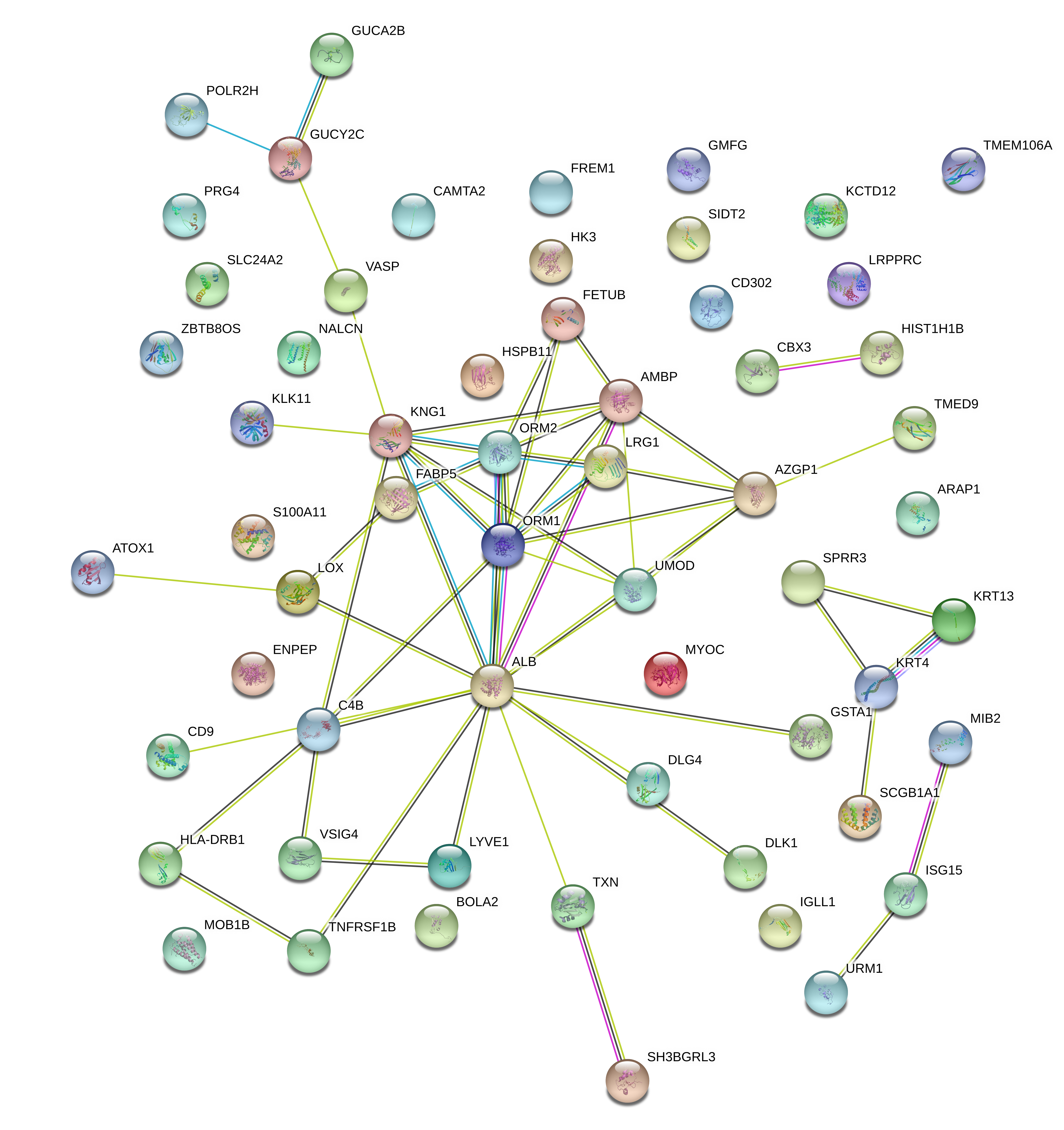

Supplement: Supplementary file 1 [file Image_1.PNG]
