# Supplementary material for: Urinary Proteomics Identifying Novel Biomarkers for the Diagnosis of Adult-Onset Still’s Disease
Source: Front Immunol. 2020 Sep 4;11:2112. doi: 10.3389/fimmu.2020.02112 (PMC7500098; doi:10.3389/fimmu.2020.02112)
Supplement: Supplementary file 3 [file Table_2.DOCX]

Supplementary Table 2. Diagnostic performance of the three urine markers for AOSD

|  | AUC | P value | Sensitivity | Specificity |
| --- | --- | --- | --- | --- |
| LRG1 | 0.700 | 0.000 | 0.928 | 0.362 |
| ORM1 | 0.837 | 0.000 | 0.783 | 0.838 |
| ORM2 | 0.736 | 0.000 | 0.580 | 0.829 |
| LRG1 plus ORM1 | 0.837 | 0.000 | 0.783 | 0.829 |
| LRG1 plus ORM2 | 0.738 | 0.000 | 0.565 | 0.829 |
| ORM1 plus ORM2 | 0.840 | 0.000 | 0.783 | 0.838 |
| LRG1 plus ORM1 plus ORM2 | 0.838 | 0.000 | 0.783 | 0.829 |

*LRG1*, alpha-1-acid glycoprotein 1; *ORM1*, leucine rich alpha-2-glycoprotein 1; *ORM2*, leucine rich alpha-2-glycoprotein 2; *AUC*, area under the curve.
