# Supplementary material for: Urinary Proteomics Identifying Novel Biomarkers for the Diagnosis of Adult-Onset Still’s Disease
Source: Front Immunol. 2020 Sep 4;11:2112. doi: 10.3389/fimmu.2020.02112 (PMC7500098; doi:10.3389/fimmu.2020.02112)
Supplement: Supplementary file 4 [file Table_3.docx]

Supplementary table 3 Comparison of the urine levels of LRG1, ORM1 and ORM2 according to the disease manifestations in AOSD patients

| Manifestations | LRG1/ORM1/ORM2 levels (ng/ml) | | |
| --- | --- | --- | --- |
|  | (+), n | (-), n | *P* value |
| Fever | 70 | 0 |  |
| LRG1 | 13.17 ±26.40 | NS | NS |
| ORM1 | 1013.00 ± 1040.00 | NS | NS |
| ORM2 | 1804.00 ± 1954.00 | NS | NS |
| Sore throat | 41 | 29 |  |
| LRG1 | 12.86 ± 27.74 | 13.60 ±24.86 | 0.26 |
| ORM1 | 804.30 ± 816.50 | 1309.00 ± 1248.00 | 0.11 |
| ORM2 | 1445.00 ± 1396.00 | 2330.00 ± 2500.00 | 0.17 |
| Skin rash | 59 | 11 |  |
| LRG1 | 13.02 ± 26.55 | 13.94 ± 26.82 | 0.24 |
| ORM1 | 981.5 ± 998.9 | 1184.00 ± 1278.00 | 0.82 |
| ORM2 | 1682.00 ± 1922.00 | 2450.00 ± 2090.00 | 0.17 |
| Lymphadenopathy | 43 | 27 |  |
| LRG1 | 16.31 ± 29.06 | 8.16 ± 21.06 | 0.04 |
| ORM1 | 1105.00 ± 1097.00 | 868.10 ± 941.90 | 0.17 |
| ORM2 | 1966.00 ± 2076.00 | 1552.00 ± 1756.00 | 0.19 |
| Hepatomegaly | 3 | 67 |  |
| LRG1 | 6.98 ± 7.81 | 13.45 ± 26.92 | 0.66 |
| ORM1 | 1305.00 ± 788.90 | 1000.00 ± 1052.00 | 0.45 |
| ORM2 | 3780.00 ± 2353.00 | 1714.00 ± 1907.00 | 0.08 |
| Splenomegaly | 21 | 49 |  |
| LRG1 | 13.98 ± 27.37 | 12.82 ± 26.25 | 0.48 |
| ORM1 | 1023.00 ± 1010.00 | 1009.00 ± 1063.00 | 0.67 |
| ORM2 | 1687.00 ± 1404.00 | 1852.00 ± 2150.00 | 0.70 |
| Pericarditis | 14 | 56 |  |
| LRG1 | 17.09 ± 2 7.08 | 12.19 ± 26.38 | 0.07 |
| ORM1 | 1308.00 ± 997.00 | 939.60 ± 1046.00 | 0.05 |
| ORM2 | 2824.00 ± 2951.00 | 1567.00 ± 1587.00 | 0.09 |
| Pneumonia | 28 | 42 |  |
| LRG1 | 19.11 ± 29.02 | 9.21 ± 24.04 | 0.01 |
| ORM1 | 1227.00 ± 1113.00 | 871.00 ± 975.50 | 0.05 |
| ORM2 | 2360.00 ± 2475.00 | 1447.00 ± 1455.00 | 0.09 |
| Pleuritis | 18 | 52 |  |
| LRG1 | 16.89 ± 28.87 | 11.88 ± 25.66 | 0.05 |
| ORM1 | 1233.00 ± 1096.00 | 937.20 ± 1019.00 | 0.06 |
| ORM2 | 2582.00 ± 2703.00 | 1550.00 ± 1592.00 | 0.14 |
| Myalgia |  |  |  |
| LRG1 | 20.43 ± 33.01 | 9.62 ± 22.02 | 0.31 |
| ORM1 | 1113.00 ± 972.50 | 946.80 ± 1078.00 | 0.33 |
| ORM2 | 2157.00 ± 1597.00 | 1628.00 ± 2105.00 | 0.06 |
| Arthralgia | 60 | 10 |  |
| LRG1 | 12.53 ± 25.07 | 16.98 ± 34.72 | 0.44 |
| ORM1 | 1058.00 ± 1097.00 | 744.90 ± 555.2 | 0.77 |
| ORM2 | 1858.00 ± 2069.00 | 1485.00 ± 1065.00 | 0.97 |

The difference between two groups were analyzed by Mann Whitney test.

*LRG1*, alpha-1-acid glycoprotein 1; *ORM1*, leucine rich alpha-2-glycoprotein 1; *ORM2*, leucine rich alpha-2-glycoprotein 2
